# Immune Assessment Today: Optimizing and Standardizing Efforts to Monitor Immune Responses in Cancer and Beyond

**DOI:** 10.3390/cancers16030475

**Published:** 2024-01-23

**Authors:** Surya Pandey, Meghan E. Cholak, Rishita Yadali, Jeffrey A. Sosman, Marie-Pier Tetreault, Deyu Fang, Seth M. Pollack, Sacha Gnjatic, Rebecca C. Obeng, H. Kim Lyerly, Adam M. Sonabend, José A. Guevara-Patiño, Lisa H. Butterfield, Bin Zhang, Holden T. Maecker, I. Caroline Le Poole

**Affiliations:** 1 Immunotherapy Assessment Core, Chicago, IL 60611, USA; surya.pandey@northwestern.edu (S.P.); meghan.cholak@northwestern.edu (M.E.C.); rishita.yadali@northwestern.edu (R.Y.); bin.zhang@northwestern.edu (B.Z.); 2 Lurie Comprehensive Cancer Center, Northwestern University at Chicago, Chicago, IL 60611, USA; jeffrey.sosman@nm.org (J.A.S.); marie-pier.tetreault@northwestern.edu (M.-P.T.); fangd@northwestern.edu (D.F.); seth.pollack@northwestern.edu (S.M.P.); adam.sonabend@northwestern.edu (A.M.S.); 3 Human Immune Monitoring Center, The Tisch Cancer Institute, Icahn School of Medicine at Mount Sinai, New York, NY 10029, USA; sacha.gnjatic@mssm.edu; 4 Case Comprehensive Cancer Center, Case Western Reserve University, Cleveland, OH 44106, USA; rebecca.obeng@case.edu; 5 Center for Applied Therapeutics, Duke Cancer Center, Duke University, Durham, NC 27710, USA; kim.lyerly@duke.edu; 6 Immune Monitoring Core, Moffit Cancer Center, Tampa, FL 33612, USA; josealejandro.guevara@moffitt.org; 7 Merck Research Laboratories, Boston, MA 02115, USA; lisa.butterfield@ucsf.edu; 8 Department of Microbiology and Immunology, University of California, San Francisco, CA 94143, USA; 9 Human Immune Monitoring Center, Stanford Cancer Institute, Stanford School of Medicine, Stanford, CA 94305, USA

**Keywords:** immune cells, biomarkers, cancer therapy, vaccine, immune monitoring, core facility

## Abstract

**Simple Summary:**

A group of experts was invited to speak at the first Immune Assessment symposium held in Chicago, IL in early October of 2023. The combined knowledge presented, including new findings presented by young investigators, offered deep insight into the importance of monitoring immune responses in (cancer) patients, the history of biomarker development and the latest technologies available to measure responses to treatment beyond clinical improvement.

**Abstract:**

As part of a symposium, current and former directors of Immune Monitoring cores and investigative oncologists presented insights into the past, present and future of immune assessment. Dr. Gnjatic presented a classification of immune monitoring technologies ranging from universally applicable to experimental protocols, while emphasizing the need for assay harmonization. Dr. Obeng discussed physiologic differences among CD8 T cells that align with anti-tumor responses. Dr. Lyerly presented the Soldano Ferrone lecture, commemorating the passionate tumor immunologist who inspired many, and covered a timeline of monitoring technology development and its importance to immuno-oncology. Dr. Sonabend presented recent achievements in glioblastoma treatment, accentuating the range of monitoring techniques that allowed him to refine patient selection for clinical trials. Dr. Guevara-Patiño focused on hypoxia within the tumor environment and stressed that T cell viability is not to be confused with functionality. Dr. Butterfield accentuated monitoring of dendritic cell metabolic (dys)function as a determinant for tumor vaccine success. Lectures were interspersed with select abstract presentations. To summarize the concepts, Dr. Maecker from Stanford led an informative forum discussion, pointing towards the future of immune monitoring. Immune monitoring continues to be a guiding light towards effective immunotherapeutic strategies.

## 1. Introduction

**Figure 1:**
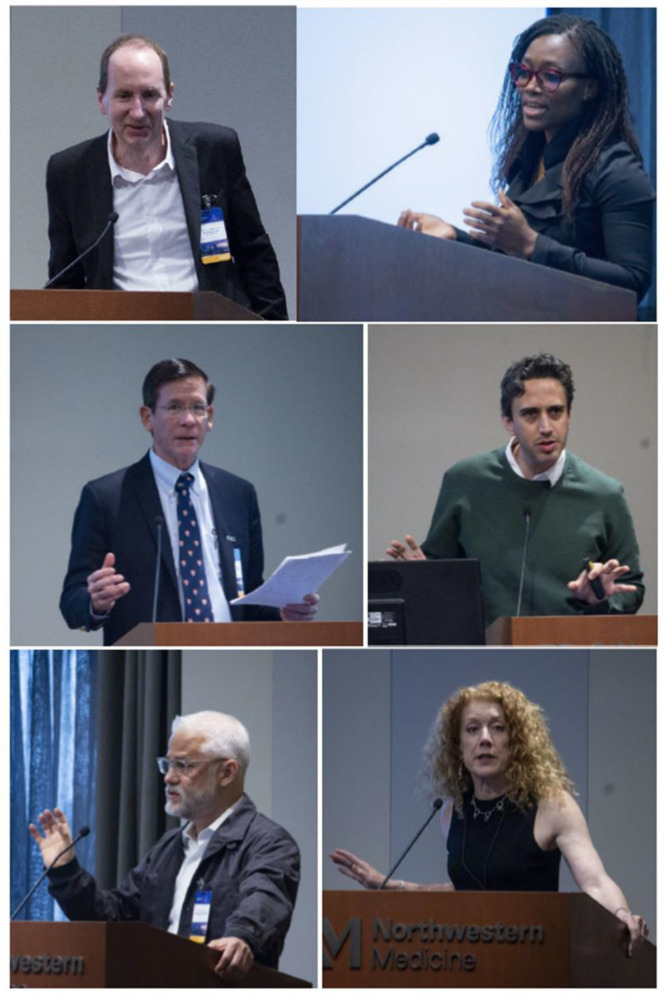
**Symposium speakers Drs. S. Gnjatic, R. Obeng, K. Lyerly, A. Sonnabend, J. Guevara-Patino, L. Butterfield.** From left to right, top to bottom: Dr. Gnjatic is a professor of Oncological Sciences, Medicine, Hematology and Medical Oncology and Pathology, and Molecular and Cell Based Medicine at Icahn School of Medicine at Mount Sinai in New York, NY, USA. Dr. Obeng is an Assistant Professor in the Department of Pathology at the School of Medicine at Case Western Reserve University in Cleveland, OH, USA. Dr. Lyerly is a Professor of Surgery, Immunology, and Pathology at Duke University School of Medicine in Durham, NC, USA. Dr. Sonabend is an Associate Professor of Neurosurgery at Northwestern University. Dr Guevara-Patiño is a Professor of Immunology at Moffit Cancer Center in Tampa, FL, USA. Dr. Butterfield is Adjunct Professor of Microbiology and Immunology at the University California San Francisco and a Distinguished Scientist for Merck.

On 5 and 6 October 2023 an Immune Assessment Symposium was held in Chicago, IL, USA. The meeting was held to promote cross-disciplinary exchanges and discuss current standards in immune monitoring and immunotherapy assessment for clinical and translational studies. The goal was to establish a forum uniting scientists studying immunotherapies and immune responses, to review the current immune monitoring techniques and establish a common framework for leveraging future technologies to precisely evaluate immunologic responses in both basic and early stage clinical research settings. To do this, we utilized the knowledge accumulated by diverse monitoring facilities equipped with cutting edge equipment to measure immune responses with a wide range of protocols. The insights gained from these facilities can be helpful for institutions nationwide and internationally when designing a repertoire of assays covering tissue imaging, cell profiling, sequencing and cytokine analytes. The goal was to align the assays with state-of-the-art measures and standardized assays performed at the National Cancer Institute (NCI)’s CIMACS or ‘cancer immune monitoring and analysis centers’ at Dana Farber Cancer Institute, Icahn School of Medicine, MD Anderson Cancer Center and Stanford University. These centers were established with support from the Cancer Moonshot initiative, that also helped support the newly instated partnership for accelerating cancer therapies or PACT. Expert speakers provided deep insight into the past, present, and future landscape of immune monitoring (Figure 1).

## 2. Multi-Omics and Cross-Trial Analyses

Dr. Sacha Gnjatic from the Icahn School of Medicine at Mount Sinai, New York was the inaugural speaker addressing the audience. His research focuses on examining human antigen-specific immune responses to tumor antigens [1], development of cancer immunotherapies [2] and characterization of the tumor-immune microenvironment [3,4,5]. At Mount Sinai he co-directs the Human Immune Monitoring Center, a vital component of the NCI-designated CIMACs. These CIMACs set standards for immune monitoring by performing high level, multiplex assays to define immunological correlates for clinical trials directed under the NCI cooperative agreements. First tier assays are recommended to be applied to all trials on longitudinally collected tumor and blood specimens, while Tier 2 and 3 assays cover more specialized technologies, allowing for deeper dives into function and mechanism in selected studies or patient subsets (Table 1). The latter assays were approved after extensive validation and may be performed ad hoc. Moving toward approval, harmonization and proficiency testing involves sharing SOPs across CIMACs, followed by interlaboratory testing and the development of a consensus SOP. At that point proficiency testing continues with intermittent group review of protocols. There is indeed a concerted effort to develop consistent biomarkers to help finetune diagnoses, predict outcomes and understand therapy responses in cancer patients. For checkpoint inhibitor therapy, FDA approved biomarkers of treatment eligibility [6] include tissue PD-L1 expression, high tumor mutation burden and/or diminished DNA damage repair, and lack of specific driver alterations. Emerging biomarkers include gut microbiome composition [7], detection of cell-free or epigenetically modified tumor DNA, and gene expression marking tumor-associated or peripheral immune cell subsets associated with treatment responses or survival. In this respect, T cells are gaining in importance, prompting an interest in understanding T cell clonality and antigen recognition, with TCR sequencing becoming critical [8]. However, we must acknowledge that innovation and standardization do not always align during assay development. Sacha further emphasized the importance of signal normalization and standardizing the process of registration, cell segmentation and deconvolution for consistent tissue staining analysis [9], and of constant inter-assay validation to support reproducibility of the results. Notably, there is growing interest in bulk analytes in serum as markers of disease improvement. Examples from CIMAC/PACT clinical trials include the predictive value of detecting IL-6 and IL-8 in serum samples from muscle-invasive bladder cancer patients, which appeared to be predictive of tumor responses to PD-1 blockade with chemotherapy [10], while reduced serum CXCL13 on treatment was often associated with better responses, including in a study of non-small cell lung cancer patients treated with combination of immune checkpoints (Parra et al., in press). These immune monitoring approaches can also be applied to correlates of adverse events associated with immunotherapies. An example is found in a study of patients developing immune checkpoint-induced colitis, a major toxicity limiting the development of drugs targeting CTLA-4 in particular. There, inflammatory signals in serum related to Th1 and Th17 pathways [11]. Overall, the implementation of well-designed, sample-rich studies assayed with harmonized multiparameter platforms should allow us to define sets of biomarkers associated with clinical benefit, allowing eventual comparisons across different treatment modalities and cancers. While this process is still in its early phase, the ultimate goal is that composite biomarkers will emerge to guide better patient selection for optimal therapies.

## 3. Biomarkers of Effective Treatment

The subsequent session focused on biomarkers of responsiveness. Arjun Kharel from the laboratory of Dr. Weiguo Cui addressed the impact of PBAF function loss [12]. Loss of PBAF was found to promote effector differentiation and limit CD8 T cell exhaustion, suggesting that the chromatin remodeling complex can be a target for cancer immunotherapy. Next, Victor Karwacinski from Dr. Daniel Brat’s lab described the connection between hypoxia and macrophage influx to tumors. This influx can promote a stem cell-like phenotype and radioresistance among glioblastoma tumors [13]. Marihan Hegazy of Dr. Kathleen Green’s laboratory showed a role for desmoglein-1 in epithelial differentiation by limiting MAPK signaling and keratinocyte proliferation, and in inflammation by suppressing TNF-induced expression of type 17 cytokines by epithelial cells [14]. William Nguyen, from Dr. Alan Zhou’s lab, shared insights on cutaneous T cell lymphoma. The data revealed distinct gut microbial profiles between controls and patients responsive to narrow-band UVB exposure, with butyrate-producing Lachnospiraceae enriched among responders [15]. In the final presentation of this session, Radhika Iyer from Dr. Deyu Fang’s group showed that ATXN3 serves as a positive regulator of PD-L1 as identified in a CRISPR screening. ATXN3 expression increased in several tumor types suggests its potential as a target for improving ICI responses [16]. Collectively, these presentations provided an excellent overview of emerging disease biomarkers with the potential for inclusion in future screening panels.

## 4. Predicting Responses to Immunotherapy

Dr. Rebecca Obeng discussed opportunities for using proliferative CD8 T cell responses in peripheral blood to predict responses to immunotherapy. As an Assistant Professor of Pathology at Case Western Reserve University, Dr. Obeng aims to understand how T cells differentiate and function within the tumor microenvironment [17]. Her work explores the spatial relationships between individual CD8 T cell subsets and elements of the tumor microenvironment, extending her research to tertiary lymphoid structures and their role in antitumor immunity. Dr. Obeng is dedicated to developing predictive bio-markers that can improve patient selection and provide a prognosis for the outcomes of cancer immunotherapy. Success metrics are informed in part by the type of therapy applied, broadly classified as B cell targeted therapy, cytokine-based treatment, adoptive T cell transfer and immune checkpoint inhibition. Initially, such predictors included evaluating immune infiltration to detect ‘hot tumors’ and measuring DNA repair activity to understand whether mutations persist to form potential neoantigens [18]. Large-scale screening now allows for the identification of biomarkers correlating with therapeutic responsiveness. While PD-L1 turned up as such a predictor molecule [19], questions remain about its relative importance when expressed by the tumor versus expression of PD-L1 by tumor-infiltrating immune cells. PD-1+ TCF-1+ Stem-like CD8 T cells are the primary targets of PD-1 checkpoint blockade, resulting in the expansion of the population and differentiation into effector cells. These responsive, stem-like cells are found in T cell-like zones within stroma-containing, tertiary lymphoid-like structures observed in several tumor types [20]. Tumor infiltration by stem-like PD-1+ TCF-1+ stem-like CD8 T cells proved to be a significant parameter of responsiveness. Early proliferative CD8 T cell responses after PD-1 blockade were predictive of positive clinical outcomes, with proliferating cells having an effector-like phenotype [21]. These findings introduce new questions, such as whether the same biomarkers are also reflective of side effects. Importantly, the transcriptional profile of the effector cells can be modified to generate better effector CD8 T cells [20], thus revealing new ways of monitoring immune responses to immunotherapy to improve clinical outcomes.

## 5. Assessment in (Pre)Clinical Trials

The discussion then shifted to examples of (pre)clinical trials where immune assessment helps to define treatment efficacy. Expanding on research initiated in the Platanias lab, Dr. Diana Saleiro showed that responses to checkpoint inhibitors are markedly enhanced by inhibiting the Unk-51 like kinase 1 (ULK1) signaling pathway. This pathway is typically involved in mediating immunosuppressive responses downstream of IFN-γ [22]. Sangeeta Kowli then discussed CyTOF data from the Maecker laboratory to identify diagnostic or prognostic factors in peripheral blood, revealing chronic activation of cytotoxic NK and T cell subsets in melanoma patients [23]. Continuing the focus on melanoma-related research, Rohan Shivde from the Le Poole lab compared outcomes for CAR T cells to bispecific T cell engagers targeting a single melanoma surface molecule. This comparison revealed differing cytokine profiles from participating T cells [24,25]. Hui Tang from Dr. Bin Zhang’s lab next described opportunities to overcome resistance to immune checkpoint blockade [26] by re-programming tumor-induced granulopoiesis through cysteinyl leukotriene receptor-1 inhibition. In the closing presentation of this session, Dr. Seth Pollack focused on the significant role of a different T cell subset. He highlighted that TLR4 agonist glycopyranosyl lipid A sensitizes the tumor microenvironment to radiation treatment, while driving clonal convergence among tumor infiltrating CD4+ T lymphocytes [27]. Tyler Smith from the laboratory of Dr. Jennifer Wu next emphasized the opportunities arising from NKG2D co-stimulation, using antibody B10G5 to target its ligand (soluble) MIC, he demonstrated enhanced stemness of CD8 T cells in tumors from a MIC-transgenic mouse model of prostate cancer [28]. April Bell from Dr. Derek Wainwright’s group revisited the topic of glioblastomas. She described how senolytics target extra-tumoral senescent cells to boost immunotherapy responses in older subjects [29].

## 6. Soldano Ferrone Lecture

Dr. Kim Lyerly was awarded the Soldano Ferrone lectureship and started his presentation by acknowledging the extensive contributions of Dr. Ferrone, who tragically succumbed to the COVID pandemic in the early days of 2023. The speaker, Dr. Lyerly is a Distinguished Professor of Immunology and a Professor of Surgery and Pathology. He is an expert in cancer immunotherapy and was a long-standing member of the NCI Cancer Advisory Board. Citing a summary by Drs. Whiteside and Zarour [30], Dr. Lyerly highlighted Soldano’s ground-breaking work on HLA class I serotyping and the identification of components of the antigen processing pathway, expression of MHC class II by tumor cells, identification of tumor antigens at the crossroads of costimulatory pathways and generating CAR T cells to target them. His passion for the field remains alive as the torch has been passed to his children [31]. Dr. Lyerly then mentioned the excitement of recognizing cytotoxic T cells as mediators of the anti-tumor response in the early days of tumor immunology [32], and the recognition that HLA-dependent cytolysis correlated with anti-tumor responses [33]. The Cancer Immunotherapy Trials Network advanced these findings further [34], starting the path to the development of peptide MHC tetramer technology [35], detecting cytokine-expressing cells by flow and performing single-cell ELISPOT analysis of cytokine expression. These methods have allowed the comparison of antigen-specific responses to anti-tumor vaccines and their correlation with clinical outcomes [36], highlighting the importance of overcoming tumor immune suppression [37]. Kim then focused on the use of virus-like replicon particles and self-replicating RNA vectors to elicit dose-dependent B and T cells responses to carcinoembryonic antigen [38,39], especially after repeated immunization. Moving to discuss immune assessment [34], CD27 has emerged as a marker of antigen-specific memory T cells recognizing Her2 in breast cancer [40], presenting opportunities to further enhance responses as a target of agonist Abs [41]. The presentation then centered around precise immune monitoring opportunities, showcasing the cancer rainbow (crainbow) mouse [42] to track HER2 isoform expression in crypts of the mouse mammary gland. In this model, the HER2 gene is randomly floxed to produce 3 different, individually labeled isoforms of the target molecule, allowing investigators to follow cellular lineage. Summarizing the current state of immune assessment, Dr. Lyerly concluded that characterizing regional and circulating immune responses to vaccines will continue to be explored at single cell and antigen specific level, while functional assays will likely remain exploratory. Dr. Lyerly underscored the importance of solid *p*-values and biologically plausible effects, validated by external results as crucial elements for future immune assessment efforts.

## 7. Refining Patient Selection

Drug delivery and refining patient selection were topics addressed by Dr. Adam Sonabend in his presentation about improving outcomes for glioma immunotherapy [43,44]. Dr. Sonabend is a brain tumor neurosurgeon/scientist specializing in the care of brain tumor patients. As an Associate Professor of Neurosurgery and Director of Translational Neuro-Oncology at Northwestern University, he has been dedicated to predictive biomarker discovery for glioblastoma immunotherapy in his research [45]. Given the immediate availability of tissues post glioblastoma diagnosis and tumor surgery, the detailed study of such predictive markers is feasible. Adam described PTEN mutations selectively associated with non-responders, whereas MAPK mutations, although very rare, are clearly associated with responses to therapy [46]. Responders consistently exhibit pERK activation and more abundant microglia [47]. Effective patient selection is critically important for this patient group with aggressive tumors, guiding them promptly to the most promising treatment options with the best odds of success. For those patients eligible for treatments like paclitaxel, Dr. Sonabend described the use of microbubbles created by an implantable ultrasound device [48]. By measuring tumor concentrations of drug in the tumor dissected after the use of microbubbles or not, the efficacy of treatment can be readily followed [49]. Brain tissue repair after ultrasound treatment is rapid and preliminary data indicate that this outcome is associated with microglia moving towards the damaged vasculature [50]. Immune assessment at the protein level has greatly helped to advance this technology in the Sonabend lab.

## 8. Measuring Hypoxia-Induced Immune Suppression

Dr. José Guevara-Patiño, Professor of Immunology and initiator of the Immune monitoring core at Moffit Cancer Center discussed research focused on signaling cues exploitable for cancer patients undergoing T cell-based immunotherapy. His research focuses on better understanding the immune fertile conditions that are necessary to generate robust anti-tumor T cell responses [51] and avoid TGFβ-driven immune suppression [52]. Currently, José is interested in understanding the role of RPS6 and the effects of tumor-related hypoxia on anti-tumor T cells [53]. By implementing machine learning approaches, he is working towards the development of immunological predictive biomarkers for stratifying cancer patients undergoing immunotherapies. José noted that patients with ECOG level 0–1 are more prone to developing immune-related adverse events [54] which, in turn, correlate with better survival. The Achilles heel for adoptively transferred T cells then lies in the hypoxic environment they encounter in the tumor [55], as this environment impedes their activity and limits their cytokine expression [56]. These T cells clearly need oxygen to survive, and they are observed in close proximity to the vasculature, ensuring close access to oxygen. It has been reported that the hypoxia signature can be tracked using pimonidazole to stain the T cells over time. This signature cannot be bypassed by non-TCR signaling and involves mitochondria that are functionally changed under hypoxia, favoring glycolysis to meet their energy needs [57]. When providing uridine to restart proliferation among hypoxic T cells, Dr. Guevara-Patiño noticed that this treatment does not re-invigorate cytokine expression.

## 9. Technical Advances

Transitioning to the topic of technical advances in the field, Dauren Biyashev, as a member of Dr. Kurt Lu’s extended research team, discussed applying synthetic melanin particles [58] as a means of scavenging radicals. This innovative approach aims to recruit anti-inflammatory immune cells and promote tissue repair, important for would healing following procedures like surgery. Anumeha Singh from the laboratory of Dr. Rui Yi described interactions between tissue stem cells and immune cells as determinants in the transcriptional control of immune privilege, taking hair follicle stem cells as an example [59]. Initiating desired immune responses can be overruled by immune privilege, and understanding the process can contribute to the design of measures to control ongoing immunity. Concluding this session, Victor Arrieta presented data from Catalina Lee-Chang’s group [60], describing integrated single cell analysis [61] of the immune landscape in chordomas, rare tumors that occur in the spine or base of the skull. In peripheral blood and tumor samples from chordoma patients, anti-tumor immunity was marked by clonal enrichment and exhaustion among both peripheral and intra-tumoral CD8+ T cells.

## 10. Monitoring Success in Cancer Vaccine Development

To better understand the benefits of cancer vaccines, Dr. Lisa Butterfield described the lessons learned to date. Professor Butterfield previously led the Immunologic Monitoring Lab at the University of Pittsburgh, and she is currently affiliated with the University of California San Francisco. Her research is focused on cancer vaccines, immune profiling and therapies for melanoma, hepatocellular cancer, and other tumor types. Her work in cancer vaccines began with pulsing HLA-A2+ patient DCs with the immunodominant non-mutated MART1-derived peptide. This work gained momentum by transducing DCs with the full-length antigen or with combinations of shared antigens in an adenovirus [62], overcoming the need for an HLA match and rendering a more universally applicable vaccine. Interestingly, the level of tumor antigen expression was not related to treatment success, whereas vaccine-induced CD8 T cell responses were significantly correlated to both progression-free survival and overall survival [63]. A quantitative increase in the frequency of antigen specific T cells in the blood made little difference for patient outcomes. IFNα was tested for its ability to enhance antitumor responses but was not successful as tested in the clinical protocol [64]. The potency measurements of DCs, including the expression of phenotypic markers and the amount of IL-12 p70 produced [65], were not predictive for in vivo immunogenicity or clinical outcome. Patient-derived DC dysfunction was identified [66], and NFκB signaling was dysregulated in melanoma patient DCs [67], affecting ICOSL and downstream T cell priming. Another critical dysregulated area of DC biology was identified as cellular metabolism [68,69]. Transcriptional profiling, population-based Seahorse assessments as well as newer single cell metabolic measures like scMEP single cell metabolic profiling and SCENITH (single cell energetic metabolism analyzed by profiling translation inhibition) were used to measure the metabolism of patient DCs. Results show that the mTOR and pAMPK pathways provide a critical regulatory node for DC. Moreover, increased glycolysis and lactate secretion were identified as markers of immune suppression in patient DC. In melanoma and hepatocellular carcinomas, DCs exhibited reduced mitochondrial functionality [70]. Emerging screening methods at the molecular level are now helping us gain a full understanding of immune dysfunction in tumor patients, setting the stage for addressing the next challenges in tumor vaccine development.

## 11. Harmonizing Immune Monitoring

**Figure 2:**
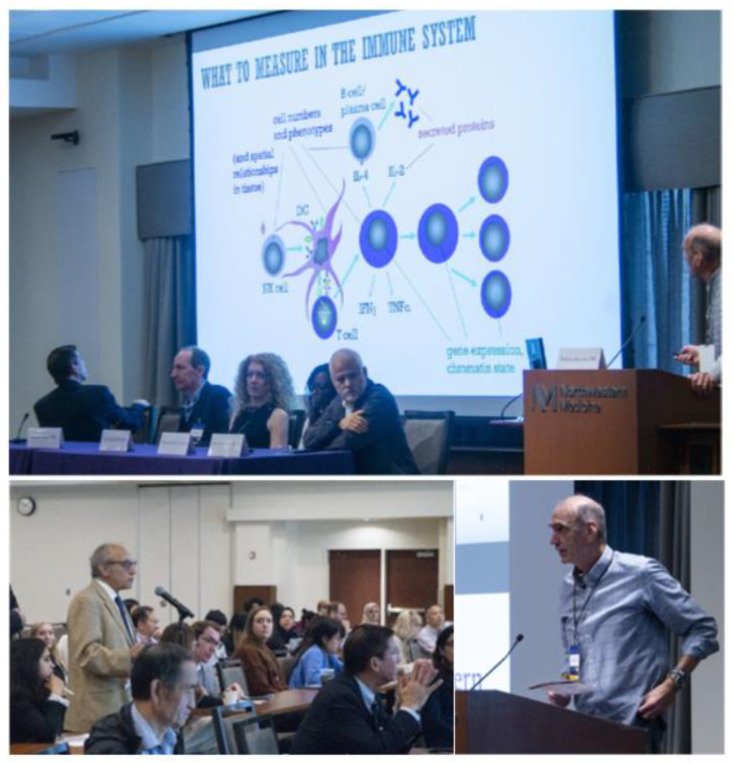
**Forum discussion and audience engagement.** (**Top image**): Forum panelists Drs. Kim Lyerly, Sacha Gnjatic, Lisa Butterfield, Rebecca Obeng and José Guevara-Patiño wondering what to measure in the immune system. Below, (**left**): Dr. Jeffrey Sosman asking a question from the audience. (**Right**): Dr. Holden Maecker leading the forum discussion.

**Figure 3:**
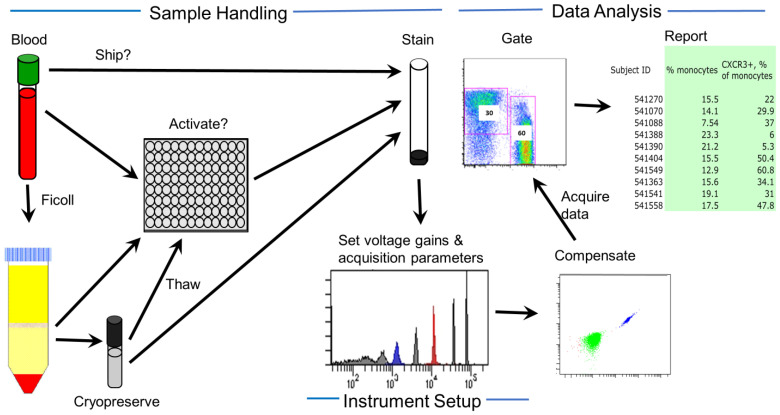
Considering sources of variability in immune assessment. Sources of variability can be classified into sample handling, instrument settings and data analysis. Sample handling variability includes the type of sample, shipping parameters, activation methods used, immune cell purification performed, freeze–thaw procedures, and staining procedures used.

With this discussion of DC and T cell characterization in tumor patients, the series of lectures morphed into a forum discussion focusing on optimal immune assessment in cancer research and treatment (Figure 2). Dr. Holden Maecker facilitated a clear discussion of methods available to cancer researchers at this time. Dr. Maecker is a Professor of Microbiology and Immunology and Director of the Human Immune Monitoring Center at Stanford University, leading one of the NCI-supported CIMACs. Holden is a member of the SITC Biomarker task force and co-chairs the FOCIS Human Immunophenotyping Consortium. His expertise defines his interest in defining metrics of immune competence and using upscale technologies to broadly survey immune features at the cellular level and link them to clinical outcomes. Holden initiated the discussion by highlighting the different parameters central to measuring immune responses, including measures of DC, NK, B cell and T cell spatial relationships, quantity and function. The initial focus was on antibodies and cytokines as the main secreted proteins to be measured, while measures of chromatin state and gene expression can give a detailed view of immune cell function. A conversation explored breakthrough monitoring technologies, parameters that cannot yet be measured, strategies to economize monitoring applications through streamlining and standardization and the role of CIMACs in guiding and unifying the field. An example discussion surrounded measurement platforms for protein analytes which include Olink, with high multiplexing capabilities, a reasonable setup mode and small sample volumes; the novel NULISA platform, which offers greater automation, with a large number of targets and a slightly larger input volume; or Luminex, with the advantage of flexibility in sample numbers and kits that are easily customized for specific uses. The sensitivity of these methods increases from Luminex to Olink to Alamar (NULISA) based on comparison testing. Another technological comparison was that of CyTOF versus spectral cytometry (Table 2), the latter allowing up to 50 marker analysis whereas spectral flow allows in the range of 40 markers to be measured. The panel design is more challenging for the latter technology with considerably more spillover, but the acquisition speed is more than 10-fold greater by spectral flow. While CyTOF does not offer light scatter parameters due to the nature of the label, spectral flow is limited by tandem dye degradation. Single cell sequencing platforms were also discussed. Among them, Mission Bio Tapestri offers targeted DNA sequencing that also allows for antibody-based proteomic measurements and is good for assessing genetic heterogeneity as relevant to tumor biology. RNA platforms including the BD Rhapsody, 10X Chromium or Fluent PIPseq and Parse Evercode all allow for sample multiplexing, while all but the Evercode offer AbSeq and CITEseq capabilities. A difference among these technologies lies in the ability to target meaningful BCR and TCR sequencing, with all but the Parse Evercode offering both and the latter offering TCR sequencing only. The features differ BD technology is suited for multiple small samples (minimum around 1000 cells) and provides the greatest cell recovery while 10X and PIPseq work with scalable cell numbers (between 10 and 200 k), and Parse Evercode allows for fixing of cells for later capture and is especially suited for high cell numbers 20 k to 1 million). The relative cost per cell is approximately 8:5:1 for BD Rhapsody:PIPseq:Parse Evercode. The discussion further extended to the sources of variability among labs, highlighting the influence of sampling, preservation, transportation methods and software selection on outcomes and reproducibility. Harmonization and standardization were deemed crucial, and it was suggested to create a single, shared analysis template, involve experts to perform QC, run comparisons with shared control samples and exclude outliers (Figure 3). Despite the recognized need to streamline monitoring strategies across core facilities, achieving full consensus remains a work in progress.

## 12. Conclusions

Data sharing and a continued discussion of Immune Assessment strategies can identify best practices, streamline clinical trial correlatives, facilitate inter-institutional comparisons, and continue to propel the field of cancer immunotherapy toward success.

## Figures and Tables

**Table 1:** CIMAC classification of immune monitoring protocols.

Tier 1	*Recommended for all longitudinally collected samples*	Whole exome sequencing RNAseq/panel-based RNA sequencing (Nanostring), PD-L1 and multiplex immunohistology for tumor tissues, TCRseq of β-chain variable regions (Adaptive), CyTOF mass cytometry Olink proximity extension assay of soluble analytes ELISA to measure immunogenicity of tumor-associated antigens from blood.
Tier 2	*Other approved assays*	scRNAseq and CITEseq Spatial transcriptomics scTCR and -BCRseq Extracellular vesicle evaluation
Tier 3	*May be performed*	Specific analysis of functional markers, phosphorylation Measurements and cytokine detection by CyTOF ELISPOT to evaluate neoantigen expression Tetramer analysis Luminex/ELLA multiplex ELISA, seromics.

**Table 2:** Monitoring by spectral flow and CyTOF: a comparison.

	Spectral Flow	CyTOF
Number of parameters	30–40	40–50
Sensitivity	Varies ~10x by channel	Varies ~4x by channel
Spillover	Significant; requires deconvolution with single-color controls, panel design can be challenging	Minimal; generally no compensation required, relatively easy panel design
Acquisition speed	3000+ cells/s	200–300 cells/s
Other limitations	Tandem dye degeneration	No light scatter parameters
